# Constructing the constitutively active ribosomal protein S6 kinase 2 from Arabidopsis thaliana (AtRPS6K2) and testing its activity in vitro

**DOI:** 10.18699/VJ20.39-o

**Published:** 2020-05

**Authors:** A.V. Zhigailov, G.E. Stanbekova, D.K. Beisenov, A.S. Nizkorodova, N.S. Polimbetova, B.K. Iskakov

**Affiliations:** M.A. Aitkhozhin Institute of Molecular Biology and Biochemistry, Almaty, Kazakhstan; M.A. Aitkhozhin Institute of Molecular Biology and Biochemistry, Almaty, Kazakhstan; M.A. Aitkhozhin Institute of Molecular Biology and Biochemistry, Almaty, Kazakhstan Institute of Plant Biology and Biotechnology, Almaty, Kazakhstan; M.A. Aitkhozhin Institute of Molecular Biology and Biochemistry, Almaty, Kazakhstan; M.A. Aitkhozhin Institute of Molecular Biology and Biochemistry, Almaty, Kazakhstan; M.A. Aitkhozhin Institute of Molecular Biology and Biochemistry, Almaty, Kazakhstan Institute of Plant Biology and Biotechnology, Almaty, Kazakhstan

**Keywords:** wheat (Triticum aestivum), S6 protein (TaRPS6) of 40S ribosomal subunits, Arabidopsis thaliana, RPS6-kinase 2 (AtRPS6K2), phosphomimetic mutation, TaRPS6 phosphorylation, пшеница (Triticum aestivum), белок S6 (TaRPS6) 40S субчастицы рибосом, Arabidopsis thaliana, AtRPS6-киназа2, фосфомиметическая мутация, фосфорилирование TaRPS6

## Abstract

Ribosomal protein S6 (RPS6) is the only phosphorylatable protein of the eukaryotic 40S ribosomal subunit. Ribosomes with phosphorylated RPS6 can selectively translate 5’TOP-(5’-terminal oligopyrimidine)-containing mRNAs that encode most proteins of the translation apparatus. The study of translational control of 5’TOP-mRNAs, which are preferentially translated when RPS6 is phosphorylated and cease to be translated when RPS6 is de-phosphorylated, is particularly important. In Arabidopsis thaliana, AtRPS6 is phosphorylated by kinase AtRPS6K2, which should in turn be phosphorylated by upper level kinases (AtPDK1 – at serine (S) 296, AtTOR – at threonine (T) 455 and S437) for full activation. We have cloned AtRPS6K2 cDNA gene and carried out in vitro mutagenesis replacing codons encoding S296, S437 and T455 by triplets of phosphomimetic glutamic acid (E). After the expression of both natural and mutated cDNAs in Escherichia coli cells, two recombinant proteins were isolated: native AtRPS6K2 and presumably constitutively active AtRPS6K2(S296E, S437E, T455E). The activity of these variants was tested in vitro. Both kinases could phosphorylate wheat (Triticum aestivum L.) TaRPS6 as part of 40S ribosomal subunits isolated from wheat embryos, though the non-mutated variant had less activity than phosphomimetic one. The ability of recombinant non-mutated kinase to phosphorylate TaRPS6 can be explained by its phosphorylation by bacterial kinases during the expression and isolation steps. The phosphomimetically mutated AtRPS6K2(S296E, S437E, T455E) can serve as a tool to investigate preferential translation of 5’TOP-mRNAs in wheat germ cell-free system, in which most of 40S ribosomal subunits have phosphorylated TaRPS6. Besides, such an approach has a biotechnological application in producing genetically modified plants with increased biomass and productivity through stimulation of cell growth and division.

## Abstract (ru)

Рибосомный белок S6 (RPS6) – единственный белок 40S субчастиц эукариотических рибосом, способный фосфорилироваться. Рибосомы с фосфорилированным RPS6 могут селективно транслировать 5’TOP (5’-terminal oligopyrimidine)-содержащие мРНК, которые кодируют большинство белков трансляционного аппарата клеток. Исследование трансляционного контроля 5’TOP-мРНК, которые преимущественно транслируются, когда RPS6 фосфорилирован, и перестают транслироваться, когда RPS6 дефосфорилируется, является особенно важным. В клетках Arabidopsis thaliana AtRPS6 фосфорилируется киназой AtRPS6K2, для активации которой, в свою очередь, требуется ее фосфорилирование киназами верхнего уровня (AtPDK1 – по серину (S) 296, AtTOR – по треонину (T) 455 и также по S437). Мы клонировали кДНК-ген AtRPS6K2 и провели его мутагенез in vitro, заменив кодоны S296, S437 и T455 на триплеты, кодирующие фосфомиметическую глутаминовую кислоту (E). После экспрессии обеих кДНК в клетках Escherichia coli были выделены два рекомбинантных белка: немутированный вариант – AtRPS6K2 и мутированный вариант – AtRPS6K2(S296E, S437E, T455E), предположительно, находящийся в стабильно активном состоянии. Активность этих киназ была протестирована in vitro. Показано, что обе киназы способны фосфорилировать рибосомный белок TaRPS6 в составе 40S рибосомных субчастиц, выделенных из зародышей пшеницы (Triticum aestivum L.), но активность нативной киназы была ниже в сравнении с ее фосфомиметической формой. Способность рекомбинантной нативной киназы фосфорилировать TaRPS6 может быть объяснена ее фосфорилированием бактериальными киназами на стадиях экспрессии и выделения. Фосфомиметически мутированная киназа AtRPS6K2(S296E, S437E, T455E) может служить удобным средством для исследо- вания избирательной трансляции 5’TOP-содержащих мРНК в бесклеточной системе из зародышей пшеницы, в которой большинство 40S рибосомных субчастиц имеет фосфорилированную форму TaRPS6. Кроме того, такой подход может найти биотехнологическое применение для создания генетически модифицированных растений с увеличенной биомассой и продуктивностью за счет стимуляции роста и деления клеток.

## Introduction

Growth and division of cells depending on the availability of nutrients, energy resources, as well as responding to internal and external stimuli are coordinated by signaling system based on a multilevel cascade of serine-threonine protein kinases. These kinases transmit signals from internal and external events to the protein synthesis apparatus, causing inhibition or enhancement of protein synthesis (Turck et al., 2004; Wolters, Jürgens, 2009; Henriques et al., 2014; Rexin et al., 2015; Roustan et al., 2016). The target of rapamycin (TOR) kinase – is the master signaling integrator, central hub synchronizing cell growth according to the nutrient and energy status as well as environmental influences (Caldana et al., 2019). In mammals, TOR forms two functionally distinct protein complexes: mTORC1 containing RAPTOR (regulatory-associated protein of mTOR), and mTORC2 containing RICTOR (rapamycininsensitive companion of mTOR) (Roustan et al., 2016). In favorable conditions mTORC1 phosphorylates RPS6K (Wolters, Jürgens, 2009; Henriques et al., 2014; Rexin et al., 2015). Complete activation of mammalian RPS6K by phosphorylation is dependent on another upper level PDK1 kinase (Otterhag et al., 2006). The fully activated RPS6K in turn phosphorylates the S6 ribosomal protein (RPS6) (Williams et al., 2003).

At transcriptional level, phosphorylation of pRPS6 in nucleolus leads to activation of rRNA gene promoter and ribosomogenesis (Ren et al., 2011; Kim et al., 2014). In cytosol, RPS6 phosphorylation promotes the selective translation of special group of cellular mRNAs, containing 5′-terminal oligo-pyrimidine tract (5′TOP) in their 5′-untranslated regions (5′UTRs) (Meyuhas, Kahan, 2015). The number of these 5′TOP-containing mRNAs, according to various estimates, ranges from one hundred to two hundred and forty (Turck et al., 1998; Meyuhas, Kahan, 2015). They encode almost all the proteins of the translation apparatus (all ribosomal proteins, all elongation factors and many of the translation initiation factors, poly(A)-binding proteins, etc.) (Turck et al., 1998), as well as other protein families associated with lysosome functions, metabolism and proliferation (Meyuhas, Kahan, 2015).

As in yeast and animals, TOR kinase is involved in controlling plant growth and cell division (Ryabova et al., 2019). But in plants, only orthologs of genes encoding mTORC1 were found (Xiong, Sheen, 2015; Wu et al., 2019). No clear orthologs of the RICTOR have yet been found in plants (Xiong, Sheen, 2015; Ryabova et al., 2019). TOR proteins are highly conserved in eukaryotes. For example, in A. thaliana and Homo sapiens they share 73 % amino acid sequence identity in the kinase domains (Xiong, Sheen, 2015).

Although functioning of this main regulator of cell processes has been well studied in other eukaryotes, knowledge of the regulation of translation and gene expression in plants is very limited. Most studies of the regulation of cellular process by plant RPS6-kinase were performed on a model object A. thaliana containing two very similar forms – At RPS6K1 and At RPS6K2. It was shown that only At RPS6K2 is able to phosphorylate RPS6 (Turck et al., 1998; Werth et al., 2019) and stimulate an increase in cell size (Rexin et al., 2015). For the complete activation of At RPS6K2, it is necessary that it be phosphorylated by pPDK1 kinase (at Ser296), pTOR kinase (at Thr455), as well as by one more, unknown, kinase (at Ser437) (Turck et al., 1998; Otterhag et al., 2006).

Although pTOR→S6K signaling plays multiple roles in translational control (Rexin et al., 2015), mechanisms used by TOR kinase to impact global protein synthesis in plants are not well understood (Xiong, Sheen, 2015; Ryabova et al., 2019; Wu et al., 2019). New data are currently appearing on the involvement of pRPS6K1 in the promotion of translation reinitiation of upstream open reading frame (uORF)-containing viral and cellular mRNAs via phosphorylation of eIF3h (Schepetilnikov et al., 2013) and in regulation of translation initiation under energy-deficient conditions via formation of the functional eIF4F complex (Lee et al., 2017). Nevertheless, the role of plant pRPS6K2 and pRPS6 phosphorylation in translation regulation in the cytosol remains unclear (Xiong, Sheen, 2015; Ryabova et al., 2019; Wu et al., 2019).

It is practically impossible to control the multiple and simultaneous phosphorylation of At RPS6K2 kinase by the kinases of the upper regulatory level for experimental purposes. Therefore, we decided to use a different approach to achieve the phosphorylation of plant RPS6 using the mutated form of At RPS6K2, which should be stably active. We have cloned the AtRPS6K2 cDNA gene and performed in vitro mutagenesis of this cDNA by replacing codons encoding serines at positions 296 and 437, as well as threonine at position 455 with triplets encoding the phosphomimetic amino acid – glutamic acid. After expression of non-mutated and mutated cDNA gene in E. coli cells the native At RPS6K2 and the phosphomimetic At RPS6K2(S296E, S437E, T455E) recombinant protein was obtained. The second one is expected to have stable kinase activity, regardless of the upper-level kinases, that could be used as a unique tool for the artificial phosphorylation of TaRPS6 in a wheat germ cell-free translation system. Mutated version of cDNA gene encoding the constantly active form of AtRPS6K2 may also be used to obtain genetically modified plants with increased productivity, earlier ripening and a higher rate of biomass accumulation.

## Materials and methods

Cloning of AtRPS6K2 cDNA gene. The total RNA was isolated from A. thaliana (Col-0 ecotype) leaves using Tri-reagent (Sigma). The reverse transcription reaction was performed using Maxima Reverse Transcriptase (Thermo) and ‘AtS6K2- rev-3UTR’ primer (5′-GAATTCGAGAAATAGGTTTCTTC AAACAACCGTTGATTTTG), which allowed to differentiate At RPS6K2 from At RPS6K1 mRNAs. RT-PCR was performed in 25 μl reaction using Phusion High-fidelity DNA polymerase (Thermo), 0.2 pM primers ‘Nde-AtS6K2-for’ (5′- GGGCGAATTGGGTCATATGGTTTCTTCTCAGTG) and ‘AtS6K2-Xho-rev’ (5′-AAACTCGAGCTACAAGTTG GATGTGGTCCGATGA) and 2.5 μl of RT-reaction mixture. Temperature regime: stage 1–5 min at 94 °C, 1 cycle; stage 2–10 s at 98 °C, 20 s at 49 °C, 45 s at 72 °С, 4 cycles; stage 3–10 s at 98 °C, 20 s at 52 °C, 45 s at 72 °С, 30 cycles; stage 4–5 min at 72 °C, 1 cycle. The PCR product (~1425 bp) was digested with NdeI/XhoI and cloned into pET19b vector digested with the same enzymes resulting ‘Pet19b-His- At RPS6K2’ plasmid.

Mutagenesis. In vitro mutagenesis was performed in three steps using QuikChange II Site-Directed Mutagenesis Kit (Agilent technologies) according to the manufacturer’s protocol. At the first step ‘Pet19b-His-AtRPS6K2’ plasmid was amplified entirely using Pfu Ultra High-Fidelity DNA polymerase (Thermo) and complementary primers: ‘S296-Glu-dir’ (5′-AAACACAAGATCAAACGAAATGTGTGGGACTA CGGA) and ‘S296-Glu-rev’ (5′-TCCGTAGTCCCACACAT TTCGTTTGATCTTGTGTTT) containing corresponding nucleotide substitutions. Temperature regime: stage 1–30 s at 95 °C, 1 cycle; stage 2–30 s at 95 °C, 1 min at 55 °C, 7 min 30 s at 68 °C, 18 cycles. The reaction mixture was further treated with restriction enzyme DpnI, which cleaves methylated DNA into fragments at 5′-Gm6ATC-3′ sequences. Since the original plasmid was methylated (dam+ E. coli strain DH5 was used for plasmid enrichment), the restriction enzyme DpnI had cleaved the original non-mutated plasmid, whereas ‘Pet19b-His-AtRPS6K2(S296E)’ plasmid synthesized during PCR-step remained intact. Subsequently, the competent E. coli cells (XL1-Blue strain) were transformed with the reaction mixture. Another two mutagenesis steps for the production of ‘Pet19b-His-At RPS6K2(S296E, S437E)’ and ‘Pet19b-His- At RPS6K2(S296E, S437E, T455E)’ plasmids were done in the same manner using ‘S437-Glu-dir’ (5′-ACATGTCTGTT TTGGATGAACCAGCAAGTAGTCCCA)/‘S437-Glu-rev’ (5′-TGGGACTACTTGCTGGTTCATCCAAAACAGAC ATGT) or ‘T455-Glu-dir’ (5′-ACCCTTTTACAAACTTCG AATACGTCAGGCCTCCTCA)/‘T455-Glu-rev’ (5′-TGAG GAGGCCTGACGTATTCGAAGTTTGTAAAAGGGT) primers respectively. Resulting DNA-constructs were used as templates for the next in vitro mutagenesis step. The inserts cloned into the recombinant plasmids were sequenced from both ends by the dideoxy chain termination method using Big Dye Terminator v.3.1 sequencing kit (Thermo) on the 310 genetic analyzer (Applied Biosystems) according to manufacturer’s recommendations.

Expression and purification of recombinant proteins. E. coli strain BL21(DE3) cells transformed with recombinant ‘Pet19b-His-AtRPS6K2’ or ‘Pet19b-His-At RPS6K2(S296E, S437E, T455E)’ plasmid were grown in 100 ml of LB medium containing ampicillin (100 μg/ml) at 30 °C to A600 of 0.5 unit. The expression of recombinant proteins was induced by 0.8 mM isopropyl β-D-1-thiogalactopyranoside (IPTG) at 30 °C for 4 h. Cells were collected by centrifugation, resuspended in His-buffer (50 mM NaH2PO4, 300 mM NaCl, pH 8.0) containing 10 mM imidazole, and then lysed by addition of lysozyme (1 mg/ml) and sonication. The cell debris was removed by centrifugation at 10000 g for 20 min at 4 °C. Supernatant was combined with PerfectPro Ni–NTA resin suspension (5-Prime), shaken at 4 °C for 1 h followed by flow throw in column. The resin was washed twice by His-buffer containing 20 mM imidazole at 4 °C. His-tagged proteins bound to the resin were eluted with His-buffer containing 250 mM imidazole and dialyzed against dialysis buffer (20 mM TrisAc, 90 mM KAc, 2.5 mM Mg(OAc)2, pH 7.6) at 4 °C for 12 h. Dialyzed proteins were concentrated by centrifugation in 10,000 MWCO HY columns (Sartorius) according to the manufacturer’s instructions. Protein concentration was estimated by the Bradford protein assay (Bradford, 1976).

Gel-electrophoresis. Proteins were separated by standard SDS-PAGE in Tris-Glycine gel system (12.5 % T, 0.5 % C separating gel; 5.2 % T, 2.5 % C stacking gel) according to U.K. Laemmli (1970). After electrophoresis, the gels were fixed and stained in PageBlue Protein Staining Solution (Thermo) or subjected to semi-dry blotting in transfer buffer (102 mM glycine, 25 mM Tris base, 20 % (v/v) ethanol) for 1 h at 0.8 mA/cm2 and 20 V using 0.22 μm pore NitroBind nitrocellulose membranes (GVS).

Western blotting. For immunodetection of His-At RPS6K2 and His-At RPS6K2(S296E, S437E, T455E) proteins, the blots were first ‘blocked’ by submerging them in blocking solution (TBST buffer (20 mM Tris-HCl; 150 mM NaCl, 0.05 % (v/v) Tween 20, pH 7.5) containing 5 % skim milk) for 1 h at 25 °C with gentle shaking. The blots were then incubated with Penta-His mouse antibodies (5 Prime) diluted (1: 2,000) in the blocking solution for 1 h at 25 °C, thoroughly washed three times with TBST buffer, and incubated for 1 h at 25 °C with horseradish peroxidase-conjugated goat anti-mouse antibodies (Santa Cruz) diluted (1: 2,000) in blocking solution. After double washes in TBST and double washes in TBS, the blots were chemiluminescence developed using Chemiluminescent Peroxidase Substrate-3 detection reagents (Sigma). An image of the membrane was then produced on X-ray film. Monoclonal Anti-Phosphoserine Mouse Antibodies (Sigma) and Monoclonal Anti-Phosphothreonine Mouse Antibodies (Sigma) were used as 1st antibodies (at 1:300 dilution in TBST containing 5 % BSA) for the detection of phosphorylation status of proteins.

40S ribosomal subunits isolation. 40S ribosomal subunits were isolated from wheat (T. aestivum L., Kazahkstanskaya-10 cultivar) embryos, purified from endosperm, as described previously for ribosomal subunits isolation from human placenta (Matasova et al., 1991) with the ratio of buffer to embryos of 6:1. It was considered that 1 A_260_ unit corresponds to 50 pmol of 40S subunits.

Kinase assay. The reaction mixture in 20 μl contained 20 mM TrisAc (pH 7.6), 90 mM KAc, 2.5 mM Mg(OAc)2, 1 mM DTT, 10 pmol of 40S ribosomal subunits, 0.1 mM ATP. Purified His-At RPS6K2 or His-At RPS6K2(S296E, S437E, T455E) were added in amount of 2.5 ng/μl. The mixtures were incubated for 20 min at 26 °C.

## Results

Cloning and mutagenesis of AtRPS6K2 cDNA gene. A total RNA preparation was isolated from A. thaliana, and reverse transcription was performed using ‘AtS6K2-rev-3UTR’ primer, complementary to 3´UTR of AtRPS6K2 mRNA, but not AtRPS6K1 mRNA, allowing to discriminate between them. Then, AtRPS6K2 cDNA was amplified by RT-PCR and cloned into pET19b vector. According to sequencing analysis, AtRPS6K2 cDNA corresponded to #AT3G08720 (GeneBank) sequence.

Thus obtained ‘Pet19b-His-AtRPS6K2’ plasmid was mutated in vitro in three steps to introduce three phosphomimetic mutations into AtRPS6K2 cDNA. At the first step, the TCC triplet encoding serine at position 296 was replaced by the GAA triplet, which encodes glutamic acid that imitates phosphorylated serine. In a second step, the TCT triplet of obtained mutated AtRPS6K2(S296E) cDNA encoding serine at position 437 was mutated to GAA triplet to form AtRPS6K2(S296E, S437E) cDNA. In the third step, the ACA triplet of AtRPS6K2(S296E, S437E) cDNA encoding threonine at position 455 was replaced by the GAA triplet to form the mutated AtRPS6K2(S296E, S437E, T455E) cDNA.

**Fig. 1.**
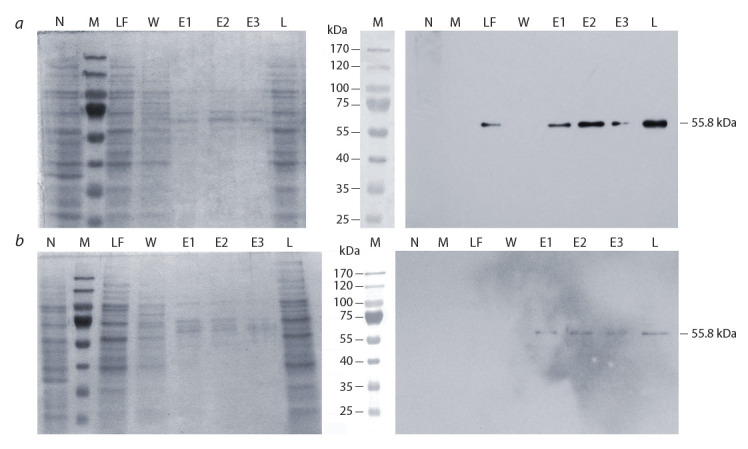
Electrophoregrams (at the left) and western blot analysis (at the right) of recombinant proteins from different fractions of IMAC chromatography. a – His-AtRPS6K2; b – His-AtRPS6K2(S296E, S437E, T455E). Left panels represent 12.5 % PAA-gels stained with Coomassie G250; right panels represent X-ray films developed after exposure with immunoblotting membranes (Penta-His Ab were used as the first antibodies). Tracks: М – protein markers; N – negative control (lysate of bacteria containing empty pET19b vector); L – lysate of bacteria synthesizing recombinant proteins; LF – proteins of wash-through fractions after loading of bacterial lysates onto Ni-NTA agarose; W – proteins eluted from Ni-NTA agarose with His-buffer containing 20 мМ of imidazole; E1, E2, E3 – proteins eluted from Ni-NTA agarose with His-buffer containing 250 мМ of imidazole.

Expression and purification of recombinant kinases. AtRPS6K2 and AtRPS6K2(S296E, S437E, T455E) cDNA genes were expressed in E. coli cells, then recombinant Histagged proteins (His-AtRPS6K2 and His-AtRPS6K2(S296E, S437E, T455E) respectively) were isolated using immobilized metal ion affinity chromatography (IMAC) followed by immunoblotting analysis (Fig. 1).

Isolated proteins were purified by dialysis and concentrated. Preparations isolated under native conditions contained a certain amount of impurity polypeptides. Content of recombinant proteins in preparations was corrected according to densitometric analysis data (by ImageJ 1.42). The yield of purified and concentrated full-length recombinant proteins His-AtRPS6K2 and His-At RPS6K2(S296E, S437E, T455E) was 5.22 mg and 4.52 mg per L of media respectively.

**Fig. 2.**
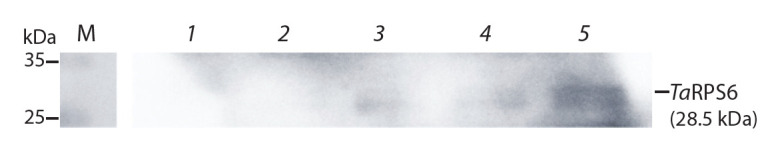
Investigation of the ability of His-AtRPS6K2 and His-AtRPS6K2(S296E, S437E, T455E) to phosphorylate TaRPS6. Monoclonal Anti-phosphoserine Mouse Antibodies diluted (1:300) in 1× TBST containing 5 % BSA were used as the 1st antibodies. All reactions contained 5 pmol of 40S ribosomal subunits isolated from wheat embryos. Tracks: 1 – negative control (without kinases); 2 – 20 ng of His-AtRPS6K2; 3 – 200 ng of His-AtRPS6K2; 4 – 20 ng of His-AtRPS6K2(S296E, S437E, T455E); 5 – 200 ng of His-AtRPS6K2(S296E, S437E, T455E); M – protein markers.

Testing the activity of recombinant kinases. Both forms of kinase (the intact one and that carrying three phosphomimetic substitutions) were tested for their ability to phosphorylate TaRPS6 in the composition of 40S ribosomal subunits isolated from wheat embryos. The phosphorylation state of proteins was tested using monoclonal antibodies against phosphoserine (Fig. 2).

As can be seen from the data presented in Fig. 2, both kinases are able to phosphorylate the plant ribosomal protein S6 (TaRPS6) in composition of 40S ribosomal subunits, although activity of His-At RPS6K2(S296E, S437E, T455E) is obviously higher than that of non-mutated His-At RPS6K2 (compare tracks 4 and 5 with tracks 2 and 3, respectively in Fig. 2). In wheat germ, there are at least two forms of the S6 ribosomal protein (A and B); therefore, two bands are observed (see e. g. track 5 in Fig. 2).

**Fig. 3.**
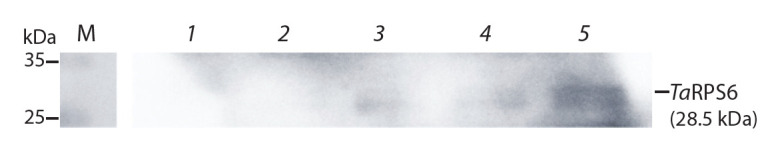
Analysis of the phosphorylation state of His-AtRPS6K2 and His- AtRPS6K2(S296E, S437E, T455E) recombinant proteins. a – analysis using monoclonal antibodies to phosphoserine; b – analysis using monoclonal antibodies to phosphothreonine. Left panels represent membranes stained with Ponso-S; right panels are immunoblots of the same membranes. Tracks: 1 – 5 μg of His-AtRPS6K2; 2 – 5 μg of His-AtRPS6K2(S296E, S437E, T455E). M – PageRuler Plus marker proteins.

Initially, we expected that non-mutated kinase should have no activity since for its activation in plant cells phosphorylation at three sites is required by upper-level kinases. The phosphorylation state of purified recombinant kinases was checked using monoclonal antibodies against phosphoserine and phosphothreonine (Fig. 3).

As can be seen from the data presented in Fig. 3, the non-mutated recombinant His-At RPS6K2 kinase produced in E. coli cells was phosphorylated both at serine residues (track 1 in Fig. 3, a) and threonine residues (track 1 in Fig. 3, b). Thus, some bacterial kinases were able to phosphorylate His-At RPS6K2 protein resulting in its activation. It should be noted that certain non-mutated serine residues of mutated His-At RPS6K2(S296E, S437E, T455E) recombinant kinase were also phosphorylated (track 2 in Fig. 3, a), although this kinase was not phosphorylated at threonine residues (track 2 in Fig. 3, b).

## Discussion

The interest in studying the mechanisms of TOR-mediated regulation of mRNA translation in plants is high because other mechanisms of regulation of protein biosynthesis, which are well described for mammals and yeast, either do not work or function within very narrow limits in plants. Indeed, in plant cells eIF4E binding proteins (eIF4E-BPs) were not found, and there are no genes for these proteins in plant genome (Immanuel et al., 2012). The mechanism of translation suppression by phosphorylation of peEF2 is not realized in plants. Then, out of four protein-kinases (PKR, HCR, PERK, GCN2) that phosphorylate α-subunit of meIF2 in mammalian cells, only pGCN2-kinase was detected in plants, that can be activated under several but not all stresses. Moreover, it was shown, that factor eIF2B is not necessary for cyclic functioning of plant peIF2 (Shaikhin et al., 1992), and neither its biochemical activity nor peIF2B-like factor orthologs were detected in plants till now (Immanuel et al., 2012). These circumstances make the TOR system one of the few currently known effective regulators of protein biosynthesis in plants.

Having obtained the constitutively active protein kinase At RPS6K2(S296E, S437E, T455E) with phosphomimetic substitutions of key amino acids, we acquire a convenient tool that allows to considerably increase phosphorylation of TaRPS6 in the composition of 40S ribosomal subunits in wheat germ cell-free system. This allows studying important mechanisms of preferential translation of a specific group of cellular 5′TOP-containing mRNAs, which is preferably translated when pRPS6 is phosphorylated and ceases to be translated when RPS6 is de-phosphorylated (Williams et al., 2003). In addition to fundamental interest the use of cDNA encoding constitutively active RPS6-protein kinase would open novel routes for increasing crop yield through stimulation of ribosomogenesis and subsequent growth and division of plant cells. It is known that augmented expression of the At TOR gene results in a dose-dependent decrease or increase, in organ and cell size, seed production (Deprost et al., 2007; Enganti et al., 2017; Bakshi et al., 2019). In addition to regulating the protein synthesis process, TOR acts as a master regulator of the cell cycle, coordinator of rRNA transcription, activation of ribosomal protein genes, ribosome assembly (Shi et al., 2018) and may also regulate long non-coding RNAs (lncRNAs) expression (Song et al., 2019). Therefore, artificial increasing of TOR gene expression in plant cells can lead to serious undesirable consequences while using of AtRPS6K2(S296E, S437E, T455E) cDNA may help to avoid these complications.

## Conclusion

We have cloned the AtRPS6K2 cDNA gene encoding kinase 2 of ribosomal protein S6 from A. thaliana and performed its mutagenesis to obtain the AtRPS6K2(S296E, S437E, T455E) kinase containing phosphomimetic substitutions. Such mutated enzyme with constant RPS6-kinase activity may be used to study specific molecular mechanisms mediating efficient translation of 5′TOP-mRNAs depending on phosphorylation of RPS6 in plant cells. At the same time, the cDNA gene AtRPS6K2(S296E, S437E, T455E) may be used to obtain genetically modified plants with increased productivity and earlier ripening.

## Conflict of interest

The authors declare no conflict of interest.
